# Hierarchical Multiple Regression Modelling on Predictors of Behavior and Sexual Practices at Takoradi Polytechnic, Ghana

**DOI:** 10.5539/gjhs.v7n4p200

**Published:** 2015-01-14

**Authors:** Anthony Joe Turkson, James Eric Otchey

**Affiliations:** 1 Department of Mathematics and Statistics, Takoradi Polytechnic, Ghana

**Keywords:** attitudes, hierarchical regressions, knowledge, sex education, sexual practices

## Abstract

**Introduction:**

Various psychosocial studies on health related lifestyles lay emphasis on the fact that the perception one has of himself as being at risk of HIV/AIDS infection was a necessary condition for preventive behaviors to be adopted. Hierarchical Multiple Regression models was used to examine the relationship between eight independent variables and one dependent variable to isolate predictors which have significant influence on behavior and sexual practices.

**Methods:**

A Cross-sectional design was used for the study. Structured close-ended interviewer-administered questionnaire was used to collect primary data. Multistage stratified technique was used to sample views from 380 students from Takoradi Polytechnic, Ghana. A Hierarchical multiple regression model was used to ascertain the significance of certain predictors of sexual behavior and practices.

**Results:**

The variables that were extracted from the multiple regression were; for the constant; *β*=14.202, t=2.279, p=0.023, variable is significant; for the marital status; *β*=0.092, t=1.996, p<0.05, variable is significant; for the knowledge on AIDs; *β*= 0.090, t=1.996, p<0.05, variable is significant; for the attitude towards HIV/AIDs; *β*=0.486, t=10.575, p<0.001, variable is highly significant. Thus, the best fitting model for predicting behavior and sexual practices was a linear combination of the constant, one’s marital status, knowledge on HIV/AIDs and Attitude towards HIV/AIDs.,

*Y* (*Behavior and sexual practices*) = *β*_0_ + *β*_1_ (*Marital status*) + *β*_2_ (*Knowledge on HIV AIDs issues*) + *β*_3_ (*Attitude towards HIV AIDs issues*)

*β*_0_, *β*_1_, *β*_2_ and *β*_3_ are respectively 14.201, 2.038, 0.148 and 0.486; the higher the better.

**Conclusions:**

Attitude and behavior change education on HIV/AIDs should be intensified in the institution so that students could adopt better lifestyles.

## 1. Introduction

Human Immunodeficiency Virus (HIV) is the virus that causes Acquired Immune Deficiency Syndrome (AIDS). AIDS has become one of the most serious health problems in the world. Since the past three decades when the virus was first identified, the body of research into the disease has been steadily growing. Today, research on the topic covers a wide range of areas ranging from medical, demographical, economical, sociological and psychological issues. These areas cover preventive, curative, and best practices that may help to halt the spread of the disease.

A greater percentage of all new HIV infections take place in Africa (UNAIDS, 2008). There is no doubt that HIV/AIDS is no longer only a public health challenge, it has a devastating impact on the country. Poverty, lack of adequate medical facilities and inadequate education are but a few of the complex factors that facilitate the spread of the disease which is undermining the hard-won economic gains of the country. The impact of HIV/AIDS is far-reaching, affecting individuals and society not only psychologically but also economically and socially. Chelala (2004) reports that although HIV/AIDS is a health problem that can be contained by effective health education programs, the deadly virus has not been contained and continues to spread so widely that it has a profound adverse impact on communities and institutions. He contends that the education sector is one of the institutions hardly hit by the virus.

HIV is transmitted through direct contact with a mucous membrane or the bloodstream with a bodily fluid containing the HIV, such as blood, semen, vaginal fluid, pre-seminal fluid and breast milk (Sepkowitz, 2001).

This transmission can involve anal, vaginal, oral sex, blood transmission, contaminated hypodermic needles, and vertical transmission during pregnancy, childbirth, breastfeeding or other exposure to one of the above bodily fluids. According to Coovadia and Bland (2007), sexual intercourse accounts for 78 percent of transmission.

The average time from infection to development of AIDS is about nine years. Thus, on the average, people do not develop AIDS until nine years after becoming infected (Zambia National HIV/AIDS/STI/TB Council 2004). For most part of this incubation period, they may not have any symptoms and therefore, may not even be aware that they are infected. This contributes to the spread of HIV since they can transmit the infection to others without knowing.

Unprotected sexual acts are riskier for the receptive partner than the insertive partner, and the risk of transmitting HIV through unprotected anal intercourse is greater than the risk from vaginal intercourse or oral sex. However, oral sex is not entirely safe, as HIV can be transmitted through both insertive and receptive oral sex. Sexual assault greatly increases the risk of HIV transmission as condoms are rarely employed and physical trauma to the vagina occurs frequently, facilitating the transmission of HIV (Koenig, 2004).

It has been established that the knowledge level of students on HIV/AIDs was fair and correlates positively with their sexual behaviors (IIiyasu et al., 2006). Similarly, Ambati et al. (1997) recognize that the knowledge level of students correlate positively with level of education. Furthermore, Serlo and Aavarinne (1999) observe that knowledge do not increase the use of safe sex but limit sexual behavior. Additionally, Odimegwu (2005) points out that knowledge and religion play an important role in sexual behavior. Hueng et al. (2005) observes that risk behavior increase with age even though knowledge increase with age. Mehra et al. (2014) believes that there is an association between poor academic performance and inconsistent condom use with a new sex partner.

Close to 60 children are raped every day in South Africa, some schools of thought are of the view that this practice culminate from the belief in the “Virgin Cure” (Earl Taylor, 2002).

This belief system, though absurd, is gaining alarming proportion in Sub-Saharan Africa. The African Traditional Religion (ATR) also holds the view that if the traditional sex laws were respected and preserved, if husbands kept to their wife or wives, if the youth avoided premarital sex, HIV/AIDS would not have come to Sub-Saharan Africa. They also hold the belief that Africans have abandoned their gods and broken the sanctity of the body, therefore the HIV disease was a punishment from the gods for sexual immorality. Finally, there is the belief that whatever has a beginning has an end, and therefore the menace of HIV will come to an end (Ekeopara et al., 2011).

Literature on health related behavior emphasize that the perception one has about being at risk of HIV/AIDS infection is a necessary condition for preventive behaviors to be put in place, what this means is that, low risk perception of the menace lead to unprotected sexual practices. Abera (1999) points out that interventions in curbing the spread of HIV/AIDS can be achieved when individuals acquire knowledge to create the desired attitude leading to behavior modification.

The 2005 Demographic Health Survey demonstrates that among those who had sexual intercourse, 1% of women and 4% of men had two or more sexual partners. 3% of women and 9% of men were involved in higher risk sexual intercourse. Also, among the sexually active youth, who engaged in high-risk sexual activity, only 25% of women and just under 50% of men used condoms in their last higher risk sexual encounter. Dintwa (2010) underscores the fact that women with primary education were less likely to be tested for HIV/AIDS and might have had sexual intercourse while intoxicated. In a study in Malaysia on sexually active girls, it was gathered that 20% had sexual intercourse with more than one partner, while 72% did not use contraceptive during the most recent sexual intercourse (Ahmadian et al., 2014).

### 1.1 The Present Study

Quite a great and impressive work have been done on HIV/AIDs and related issues, these works range from tracing the history and origin of the disease, to establishments of connections between sets of the variables; knowledge, beliefs, attitude, behaviors and practices, further studies have been done on monitoring, preventive measures, and care and cure of people living with HIV/AIDs. Others have assessed the prevalence rates of the disease within regions in the world and communities. Indeed, one can say without any shadow of doubt that the disease has become a global decimator of humankind and thus requires such urgency from researchers.

It is an established fact that knowledge about HIV/AIDs does correlate positively with sexual practices though some authors argue differently. Belief systems also have some degree of association with sexual practices, these belief systems must not be left uninvestigated.

Observational studies made at Takoradi Polytechnic on the indicators of the students’ knowledge level on HIV/AIDS vis-à-vis their latent practices are highly suspicious making them vulnerable and open to more risky behaviors. Though the youth is the greatest hope for turning the tide, they highly remain the center of the HIV/AIDS epidemic in terms of rate of infection, impact and potential for change. They have grown up in a world tainted by HIV/AIDS. Their attitude towards the disease and their practical approach to curbing the spread of the disease also leave much to be desired. It is for this perceived ignorance, their lackadaisical attitude and risky practices that necessitated this study. The objectives of the study were;

- To identify the predictors of behavior and sexual practices
- To model the relationship between independent variables and sexual behavior and practices of students towards HIV/AIDS.

In carrying out the objectives set out in this study, certain hypotheses were formulated and tested. The following were four of the hypotheses;

*Hypothesis one*: Students sexual practice (Abstinence, Being faithful to one partner and Condom usage [ABC]) is independent on their level of knowledge about HIV/AIDS.

*Hypothesis two:* Students behavior and sexual practices is independent on gender

*Hypothesis three:* There is no relationship between marital status of person and sexual practices.

*Hypothesis four:* There is no relationship between student’s belief system and sexual practices.

The importance of this study cannot be overemphasized; firstly, it unearths the knowledge base of the students about HIV/AIDS; secondly, it isolates the predictors of behavior and sexual practices. Finally, it provides stakeholders of Polytechnic Education enough grounds upon which preventive and healthy lifestyles would be inculcated into the students.

## 2. Method

The study population consisted of all students of Takoradi Polytechnic. The student population as at November 2012 was 8779. More than half of the student population was female. The city in which the Polytechnic is situated is rich in Gold, Oil, Gas, Timber, and Rubber. The city prides itself with a Port and harbor and an airport. Various activities and businesses are booming in the city.

Cross- sectional design was used to conduct the study. Data on Takoradi Polytechnic student’s knowledge, attitude and practices towards HIV/AIDS was thoroughly investigated. Data was collected between November 22^nd^, 2012 and November 29^th^, 2012. The methodological considerations were well planned and executed, the relationships between variables were studied carefully, correlation and causation relationships were carefully distinguished.

The main data collection instrument used was a closed-ended questionnaire. The questionnaire was in five sections;

*Section A* dealt with the demographic characteristics of the students.

*Section B* covered the level of knowledge of students about HIV/AIDS issues.

*Section C* covered the belief systems of students towards HIV/AIDS.

*Section D* covered the attitudes of students towards HIV/AIDS. 

*Section E* looked at the behavior and sexual practices students have adopted regarding HIV/AIDS.

A pilot study was conducted to assess the reliability of the instrument. In order to obtain a sample size which was representative of the population at a confidence level of 95 percent and a margin of error of 5 percent, the formula below was used;


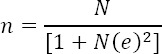


Where *n*= the sample size;

N= total student population;

*e*=margin of error of 5%.

Again, to maintain the relative proportion of the students by school (faculty), the total student population was stratified according to the four schools using the formula,


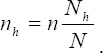


Where *n_h* is the sample drawn from “school *h”, h is the stratum number*; *n* is the total sample drawn from all the strata (schools); *N_h* is the total population for stratum *“h”* and N is the total population.

The sample sizes collected from School of Business and Management Studies (SOBAMS), School of Applied Science (SAS), School of Applied Arts (SAS) and School of Engineering (SOE) were 223, 23, 71 and 66 respectively.

The number of students from each school was further stratified according to programs offered in the school, in other words, the researcher adopted the multistage sampling technique. In the school of business for instance, students from Accountancy, Purchasing, Marketing, Secretaryship and Tourism departments were included. Due to the sensitivity of the questions, some students felt reluctant to complete the questionnaire, and as such the quota sampling technique was adopted at a point in time. In this case, sampling units were selected according to their readiness to complete the questionnaire. The main objective for using this method aside the challenges was to select a sample that will represent the major characteristics of the students from each of the programs within the school.

Permission was sought from each of the would-be-respondents; those who accepted the terms and conditions were asked to complete the questionnaire; those who thought otherwise were allowed to leave without completing the questionnaire. Each student was encouraged to be truthful as far as the responses to the questions were concerned. No student was asked to write down his or her name.

Responses for each of the four variables; Knowledge, Attitude, Beliefs and Practice were then summed up. The score ranged from 14 to 70 for all the four variables, a score of 70 on the knowledge scale indicated that the knowledge level on HIV/AIDS issues was high, a score of 14 meant the level of knowledge was very poor. On the attitude scale, a score of 70 represented a positive attitude towards the HIV/AIDS menace while a score of 14 showed a negative attitude towards the disease. On the belief system scale, a score of 70 represented a strong belief system towards HIV/AIDS while a score of 14 meant a lesser belief system towards the disease. Finally on the behavior and practices scale, 14 represented a risky practice while a score of 70 represented a risk free practice. These scores were keyed into the Statistical Package of Social Science (SPSS) version 17 and analyzed using the Hierarchical Multiple Regression Analysis.

The model for the study is given as;


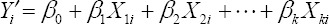


The *β* values are called regression weights and are computed in a way that minimizes the sum of squared deviations;


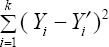


In this model there were K predictor variables rather than two and K+1 regression weights which were estimated, one for each of the K predictor variable and one for the constant (*β*_0_) term (Stockburger, 2003).

A group of variables were controlled. Firstly, multiple regression analysis was performed with the controlled variables serving as independent variables. A second multiple regression was done with a new set of independent variables together with the first step independent variables. This allowed estimates of the contributions of the independent variables to be computed. The process was continued until all the independent variables had been entered into the regression model.

## 3. Results

From Table 1, we could see that majority 95.3% of the respondents were Christians, 3.7% were Muslims, while ‘Other’ religions constituted 1.0%. Again, 39.7% of the respondents were between the age group 18-25years, 45.8% were between the age group 26 to 33 years, while 14.5% were above 34 years.

**Table 1:** Socio-demographic Profile of Respondents

Explanatory variables	Frequency	Percentage
***Gender***		
Male	198	52.1
Female	182	47.9
***Age***		
18-25	151	39.7
26-33	174	45.8
34 and above	55	14.5
***Marital status***		
Married	8	2.0
Single	328	86.2
Attached	44	11.8
***Employment status***		
Employed	11	2.9
Self employed	22	5.8
Unemployed	347	91.3
***School/Faculty***	347	91.3
Applied Arts	75	19.7
Applied Science	23	6.1
School of Business	222	58.4
School of Engineering	60	15.8
***Religious Affiliation***		
Christians	362	95.3
Moslems	14	3.7
Others	4	1.0

Source: field work 2013.

It was also noted that out of the respondents, 52.1% were males, while 47.9% were females. In addition, 86.2% of the respondents were single, 11.7% were attached, and 2.1% were married. 19.7% of the students were from School of Applied Arts, 58.4% from the School of Business, 15.8% from the School of Engineering and 6.1% from the School of Applied Science. Finally, 2.9% were employed, 5.8% were self-employed, while 91.3% were unemployed.

To ascertain whether the Hierarchical Multiple Regression model could be used for the study and to ensure the validity of the data, some first line test were conducted. Firstly, the sample size of 380 was deemed adequate given the number of independent variables (8) subjected to the test. The ratio for this analysis was 380 valid cases to 8 independent variables.

This boiled down to 48 to 1, which satisfies the minimum requirement of 15 to 1 (Tabachnick & Fidell, 2001). The assumption of singularity was also met, a critical look at Table 2 shows that none of the independent variables (Knowledge, Attitude, Beliefs, Age, Marital status, Employment status, Gender and Religious Affiliation) correlated highly with each other. For the problem of multi-collinearlity to be encountered, tolerance has to be close to zero while variance inflation factor (VIF) has to be close to 10, but that was not the situation as revealed in Table 4.

**Table 2:** Model Summary of Hierarchical Multiple Regression

Model	R	R^2^	Adjusted R^2^		∆R^2^	∆F	df_1_	df_2_	Sig. ∆F
1	.168^a^	.028	.023		.028	5.463	2	377	.005
2	.180^b^	.033	.022		.004	.852	2	375	.427
3	.208^c^	.043	.030		.011	4.119	1	374	.043
4	.307^d^	.094	.080		.051	21.007	1	373	.000
5	.561^e^	.315	.302		.221	119.886	1	372	.000
6	.563^f^	.317	.302		.002	1.146	1	371	.285

Notes:

a. Predictors: (constant), age of respondent, gender;

b. Predictors: (constant), age of respondent, gender, religious affiliation, employment status;

c. Predictors: (constant), age of respondent, gender, religious affiliation, employment status, marital status;

d. Predictors: (constant), age of respondent, gender, religious affiliation, employment status, marital status, knowledge about aids;

e. Predictors: (constant), age of respondent, gender, religious affiliation, employment status, marital status, knowledge about aids, attitude towards HIV/AIDS;

f. Predictors: (constant), age of respondent, gender, religious affiliation, employment status, marital status, knowledge about aids, attitude towards HIV/AIDS, beliefs about HIV/AIDS;

g. Dependent Variable: Behavior and sexual practices.

**Table 3:** ANOVA Results of the Six - Model- Hierarchical Regression Analysis

Model		Sum of Squares	df	Mean Square	F	Sig.
**1**	**Regression**	924.038	2	462.019	5.463	.005^a^
	**Residual**	31883.738	377	84.572		
	**Total**	32807.776	379			
**2**	**Regression**	1068.276	4	267.069	3.155	.014^b^
	**Residual**	31739.501	375	84.639		
	**Total**	32807.776	379			
**3**	**Regression**	1414.004	5	282.801	3.369	.005^c^
	**Residual**	31393.772	374	83.941		
	**Total**	32807.776	379			
**4**	**Regression**	3087.809	6	514.635	6.459	.000^d^
	**Residual**	29719.967	373	79.678		
	**Total**	32807.776	379			
**5**	**Regression**	10331.375	7	1475.911	24.427	.000^e^
	**Residual**	22476.402	372	60.420		
	**Total**	32807.776	379			
**6**	**Regression**	10400.619	8	1300.077	21.526	.000^f^
	**Residual**	22407.157	371	60.397		
	**Total**	32807.776	379			

**df** means degrees of freedom; **F** is the calculated value of the Analysis of Variance (ANOVA).

**Table 4:** Summary of Hierarchical Regression analysis for variables predicting Behavior and sexual practices

Model		B	Beta	t	Sig	Tolerance	VIF
**1**	**(Constant)**	51.257		26.356	0.000		
	**Gender**	3.031	0.163	3.205	0.001	0.997	1.003
	**Age of respondent**	0.668	0.050	0.978	0.329	0.997	1.003
**2**	**(Constant)**	55.265		12.385	0.000		
	**Gender**	3.004	0.162	3.174	0.002	0.997	1.003
	**Age of respondent**	0.672	0.050	0.982	0.327	0.995	1.005
	**Religious affiliation**	-1.961	-0.063	-1.229	0.220	0.990	1.010
	**Employment status**	-0.657	-0.028	-0.554	0.580	0.989	1.011
**3**	**(Constant)**	50.931		10.330	0.000		
	**Gender**	2.554	0.137	2.638	0.009	0.944	1.059
	**Age**	0.679	0.051	0.997	0.319	0.995	1.005
	**Religious**	-1.973	-0.063	1.242	0.215	0.990	1.010
	**Employment status**	0.094	0.004	0.076	0.939	0.901	1.110
	**Marital status**	2.440	0.110	2.029	0.043	0.864	1.157

**4**	**(Constant)**	32.566			5.206	0.000		
	**Gender**	2.385	0.128	2.527	0.012	0.943	1.061
	**Age of respondent**	0.644	0.048	0.970	0.333	0.995	1.005
	**Religious affiliation**	-2.154	-0.069	-1.391	0.165	0.989	1.011
	**Employment status**	-0.318	-0.014	-0.263	0.793	0.896	1.117
	**Marital status**	2.497	0.113	2.132	0.034	0.864	1.157
	**Knowledge on aids**	0.374	0.227	4.583	0.000	0.992	1.008

**5**	**(Constant)**	17.070			3.033	0.003		
	**Gender**	1.359	0.073	1.643	0.101	0.931	1.074
	**Age of respondent**	0.945	0.070	1.633	0.103	0.993	1.007
	**Religious affiliation**	-0.982	-0.031	-0.726	0.468	0.983	1.017
	**Employment status**	-0.663	-0.029	-0.630	0.529	0.895	1.118
	**Marital status**	2.020	0.091	1.979	0.049	0.863	1.159
	**Knowledge about aids**	0.154	0.093	2.086	0.038	0.918	1.089
	**Attitude towards AIDS**	0.495	0.495	10.949	0.000	0.900	1.111

**6**	**(Constant)**	14.202		2.279	0.023		
	**Gender**	1.342	0.072	1.623	0.106	0.931	1.075
	**Age of respondent**	0.915	0.068	1.581	0.115	0.991	1.010
	**Religious affiliation**	-0.953	-0.031	-0.705	0.481	0.983	1.017
	**Employment status**	-0.629	-0.027	-0.597	0.551	0.894	1.119
	**Marital status**	2.038	0.092	1.996	0.047	0.862	1.160
	**Knowledge on aids**	0.148	0.090	1.999	0.046	0.913	1.095
	**Attitude towards Aids**	0.486	0.486	10.575	0.000	0.870	1.149
	**Beliefs about AIDS**	0.075	0.047	1.071	0.285	0.947	1.056

Dependent variable: Behavior and sexual practices.

This implies that there was no multi-collinearlity (Coakes, 2005). A six stage Hierarchical Multiple Regression was conducted to examine the relationship between the set of independent variables; knowledge, attitude and beliefs against the dependent variable behavior and sexual practices after controlling for the effects of age, marital status, employment status, gender, and religious affiliation. From Table 2 (that is, Model 1 with age and gender as predictors of behavior and sexual practices), the R value was 0.168, thus, a positive relationship existed between the predictor variables and behavior and sexual practices, though the relationship was weak.

**Figure 1:**
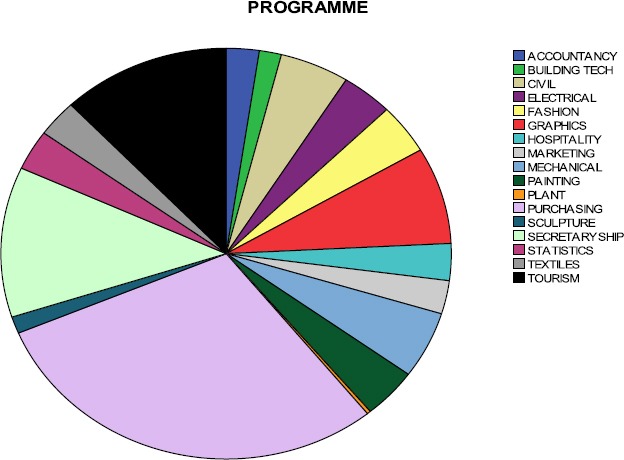
A Pie chart showing the distribution of students to the various programmes and the proportion of students from each programme

The R^2^ (0.028 or 2.8%) was significant at *F* (2, 377) = 5.463, p < 0.05, since it could account for 2.8% of the variance. Model 2, with four predictor variables (age, gender, religious affiliation and employment status), was an improvement over the earlier model, with an R of 0.180 and an R^2^ change of 0.033, thus 3.3% of the variance had been accounted for. The change in R^2^ was not significant *F* (2,375), p > 0.05; this shows that the second set of predictors (religious affiliation and employment status) could not predict behaviour and sexual practices. Model 3, with five predictor variables (age, gender, religious affiliation, employment status and marital status), gave a better value for R= 0.208, with an R^2^ of 0.043, thus 4.3% of variance was accounted for. The change in R^2^ was significant *F* (1, 374) = 4.119, p < 0.05, thus marital status was a predictor of behavior and sexual practices. Model 4, with six predictor variables (age, gender, religious affiliation, employment status, marital status, and knowledge), was quite better, with an R of 0.307 and an R^2^ of 0.094, thus 9.4% of the variance had been accounted for, the change in R^2^ was highly significant *F* (1,373) = 21.007, p < 0.001, consequently knowledge on HIV/AIDs issues had been isolated as a predictor of sexual behaviors. Model 5, with seven predictor variables (age, gender, religious affiliation, employment status, marital status, knowledge and attitude towards HIVAIDs issues), escalated the R value from 0.307 to 0.561, with an R^2^ value of 0.315, thus 31.7 % of the variance had been accounted for. The R^2^ change was also highly significant *F* (1, 372) = 119.886, p < 0.001. This results clearly showed that attitude towards HIV/AIDs contributed significantly towards behavior and sexual practices. The sixth and final model comprised of eight predictor variables (age, gender, religious affiliation, employment status, marital status, knowledge, attitude towards HIVAIDs issues and belief on HIV/AIDs issues), gave an R value of 0.563 and an R^2^ of 0.317, again 31.7% of the variance had been accounted for. The R^2^ change was not significant *F* (1, 371) =1.146, p>0.05. Thus one’s belief systems on HIV/AIDs issues do not have any significant effect on behavior and sexual practices.

The ANOVA result (Table 3) gave us the significance of each of the six models (two predictors, four predictors, five predictors, six predictors, seven predictors and eight predictors respectively). It could be seen that all six models were significant; (p < .05, p <.05, p <.05, p < .001, p < .001 and p < .001 respectively). It was noted in particular that the *F* value was largest for the model with seven predictors. The *F* values were the overall predictive effects which were different from the *F* for the amount of change experienced when adding an additional variable.

From Table 4, the *β* coefficients for the constant and eight predictors of behavior and sexual practices were as follows; Constant *β* =14.202, t =2.279, p=0.023: significant; Gender, *β* = 0.072, t=1.623, p=0.166: not significant; Age, *β* =0.068, t=1.581, p=0.115: not significant; Religious affiliation, *β* =-0.031, t=-0.075, p=0.481: not significant; Employment status, *β* =-0.027, t =-0.595, p=0.511: not significant; Marital status, *β* =0.092, t=1.996, p<0.05: significant; Knowledge on AIDs, *β* =0.090, t=1.996, p<0.05: significant; Attitude towards HIV/AIDs, *β* =0.486, t=10.575, p<0.001: highly significant; Beliefs about HIV/AIDS, *β* =0.075, t=1.071, p=0.285: not significant.

The best fitting model for predicting behavior and sexual practices from the analysis above would be the linear combination of the constant, marital status of the person, knowledge on HIV/AIDs and Attitude towards HIV/AIDs.

*The Model*

*Y* (*Behavior and sexual practices*) = *β*_0_ + *β*_1_ (*Marital status*) + *β*_2_ (*Knowledge on HIV AIDs issues*) + *β*_3_ (*Attitude towards HIV AIDs issues*)

Where, *β*_0_, *β*_1_, *β*_2_ and *β*_3_ are respectively 14.201, 2.038, 0.148 and 0.486.

## 4. Discussion

Several studies have linked the relationship between knowledge and attitude as predictors of sexual practices (Abera, 1999; Jacob et al., 2004; Serlo, 1999). The same revelation is being held by this study. The study revealed that student’s sexual practices is dependent on their level of knowledge on HIV/AIDs and related issues. It also depends on the gender of the person, moreover, one’s marital status has to a larger extend an effect on sexual practices. It was however noted the belief system held by an individual on HIV/AIDs issues has no effect on sexual practices. Even though some schools of thought were of the view that risk behavior increased with age (Hueng et al., 2005), this study could only affirm that there was a weak relationship between age and sexual practices. A disturbing phenomenon that is running throughout Africa in general and Ghana in particular is the notion of the virgin cure of HIV/AIDS, countless number of older men with HIV infections prefer to go in for young adolescent females with the view of curing their infection, it is not surprising to note that more adolescent females are affected or infected with this pandemic than their male counterparts of similar ages. It is worthwhile to impress upon the adolescent female to be wary of gifts that are been showered on them by the elderly men. Serlo and Aavarinne (1999) averred that religion played an important role for sexual behavior, that assertion was also supported in this study; indeed religion plays a significant role in the life of individuals in any society. Its role as a moral builder has been variously acknowledged (Odimegwu, 2005). Religious views on sexual behavior of persons without partners have always been outright ban, discouragement and neutrality. We should therefore uphold the sanctity of sex and strongly encourage the young ones to engage in sexual practices only in a loving, and legally committed relationships. Furthermore, the assertion that female students were sexually active than their male counterparts was upheld by this study which revealed that there was a correlation between gender and sexual practices. Female students demonstrated a higher level of knowledge in comparison to their male counterparts irrespective of the fact that there were more females in the study than males. The dominant predictor of sexual practices among students of the Takoradi Polytechnic was attitude towards HIV/AIDs and related issues, this study confirms the findings of Abera (1999) who pointed out that interventions in curbing the spread of HIV/AIDS could be achieved when individuals acquire knowledge to create the desired attitude leading to behavior modification. Thus, it is required that students of the Takoradi Polytechnic demonstrate a positive attitude towards the disease, in so doing their behavior and sexual practices will be improved. The question one may ask at this point in time is how can these students improve upon their attitudes? Firstly, there is the need to change their perspective about the disease, to date many people have negative attitudes towards PLWHA. Secondly, many people think that the disease is far away from them, and that there is no way they can contract it. Thirdly, many people have the erroneous impression that the disease can only be contracted through sex, forgetting about the fact that they could contract it though sharing razors, sharp knives and brushes with colleagues who have been infected but who show no symptom of the disease since the sickness has not yet manifested itself. These and many others are enough grounds upon which people should change their attitude towards the disease. The study also revealed that one’s knowledge on HIV/AIDS and related issues affects one’s sexual practices. It is therefore incumbent upon stakeholders of higher education institutes to include in the teaching curriculum studies on HIV/AIDS. Though some schools of thought are of the view that better knowledge does not necessarily lead to change in behavior, it is our believe that when all stakeholders get on board and address this important subject, chances are that a certain percentage of the student population will acquire additional knowledge on the deadly disease and adopt better sexual practices. The last but not the least factor as revealed in the study has to do with the marital status of the students. In this regard, the necessary steps must be taken to step down the tide by encouraging students to abstain or in the worst case scenario to use condoms as they await their turn to climb the marital ladder.

## 5. Conclusion

At the onset, we set out to use the Hierarchical Multiple Regression Model to examine the relationship between a set of eight independent variables (age, gender, religious affiliation, employment status, marital status, knowledge, attitude towards HIVAIDs issues and belief on HIV/AIDs issues) and a dependent variable (behavior and sexual practices) to isolate the predictors which have significant influence on behavior and sexual practices. We were also to model the relationship among the variables; knowledge, beliefs, attitude, and sexual practices of students towards HIV/AIDS.

A Cross-sectional design was used for the study. A structured close-ended interviewer-administered questionnaire was used to collect primary data. Multistage stratified technique was used to sample views from 380 students from Takoradi Polytechnic, Ghana. Six stage Hierarchical multiple regression models were used to ascertain the significance of certain predictors on sexual behaviors and practices. At the sixth and final stage, it was gathered that the best fitting model for predicting behavior and sexual practices was a linear combination of the constant factor, marital status of the person, knowledge on HIV/AIDs and Attitude towards HIV/AIDs.

On account of the above revelation, all the stakeholders of health education are being called upon to help minimize the menace of the HIV/AIDS disease. We note in particular that knowledge about HIV/AIDs issues and attitude of students towards the disease were the significant predictors of practices and sexual behaviors, on that note, we call on the following;

### 5.1 Students

HIV/AIDs risk behaviors among students include, unprotected sex, commercial sex work, injection of drugs, homosexuality and lesbianism. All students are therefore called upon to be wary of the seeming benefits that they hope to derive from the aforementioned activities; they are to note in particular that these activities have the potential of driving them to their early grave. There should also be peer education and role plays by PLWHA. Quizzes and debates on HIV/AIDs issues must also be intensified in the institution to help step down their negative attitudes.

### 5.2 Health Providers

The provision of HIV/AIDs information should be done with the seriousness and tenacity it deserves to all health seekers. The providers must also organize AIDs fora with the view of disseminating information.

### 5.3 Barbers and Beauticians

They must be educated to know how to prevent the transmission of the disease from an infected person to an uninfected person.
